# Influence of the Heat Transfer Process on the Electrical and Mechanical Properties of Flexible Silver Conductors on Textiles

**DOI:** 10.3390/polym15132892

**Published:** 2023-06-29

**Authors:** Tomasz Raczyński, Daniel Janczak, Jerzy Szałapak, Sandra Lepak-Kuc, Dominik Baraniecki, Maria Muszyńska, Aleksandra Kądziela, Katarzyna Wójkowska, Jakub Krzemiński, Małgorzata Jakubowska

**Affiliations:** 1 Institute of Metrology and Biomedical Engineering, Faculty of Mechatronics, Warsaw University of Technology, 00-661 Warsaw, Poland; 2 Centre for Advanced Materials and Technologies (CEZAMAT), Warsaw University of Technology, 02-822 Warsaw, Poland

**Keywords:** screen-printing, heat transfer, flexible electronics, textile electronics, wearable electronics

## Abstract

With the increase in the popularity of wearable and integrated electronics, a proper way to manufacture electronics on textiles is needed. This study aims to analyze the effect of different parameters of the heat transfer process on the electrical and mechanical properties of flexible electronics made on textiles, presenting it as a viable method of producing such electronics. Wires made from different composites based on silver microparticles and an insulating layer were screen-printed on a release film. Then, they were transferred onto a polyester cloth using heat transfer with different parameters. Research showed that different heat transfer parameters could influence the electrical properties of screen-printed wires, changing their resistance between −15% and +150%, making it imperative to adjust those properties depending on the materials used. Changes in the settings of heat transfer also influence mechanical properties, increasing adhesion between layers at higher temperatures. This study shows the importance of tailoring heat transfer properties and the differences that these properties make.

## 1. Introduction

The wearable electronics market has experienced significant growth in recent years, reaching over USD 120 billion in 2021 and being expected to surpass USD 390 billion by 2030 [1]. The market encompasses a wide range of products, from smart bands, smart watches, and pulsometers to more advanced medical applications such as glucose monitors. Currently, such solutions are offered only as stand-alone devices. However, in recent years there has been an increased interest in developing such devices integrated with clothing [2]. The integration would enable a broader range of sensors [3,4,5] that could be used for daily health monitoring or remote analysis in telemedicine. Textile-integrated electronics could be discreetly hidden underneath the clothing to ease the everyday use of such devices or be prominently displayed as a fashion statement.

Printed electronics are highly promising for such applications [6]. They offer reproducibility, reliability, and mass production at a low cost [7] and provide many unique advantages, such as the ability to print in different shapes and sizes with various specialized materials and nanomaterials [7,8,9], as well as flexibility and lightweight designs. There are multiple printing processes, including inkjet printing, spray painting, roll-to-roll printing, stencil printing, spin coating, and dip coating [6], but screen printing is considered the most promising [2,10,11]. 

There were many attempts at conventionally screen printing electronics by printing directly on textiles [5,12,13,14,15,16]. However, printing on a textile comes with distinct challenges. Due to the complicated structure of a woven textile substrate, there are some issues that do not appear when printing on a foil. The woven substrate is very porous and soaks up printing inks, causing changes in print geometry and thickness that lead to a loss of repeatability [12]. In addition, most applications of flexible electronics consist of multilayered structures [5,16] and due to the pliability of textiles, positioning each layer is impractical for large-scale production. To overcome those issues, a new variation of the screen-printing process was proposed [8,17,18,19,20]. The screen-printing process is augmented by a heat transfer process, either by heat pressing polymer ink on a textile to improve it as a substrate [17] or by printing a whole application on a release film and then transferring it [8,18,19,20]. Research shows that the heat transfer process impacts the electric properties of printed materials [20], so there is a need to explore changes that can happen properly. There are a few works to be found in the literature that focuses on the mechanical and electrical properties of heat transfer electronics [19]. Nevertheless, it is still limited and difficult to find any studies that include an analysis of the influence of parameters of the heat transfer process on the finished prints.

This research work is designated to study the effect of different heat transfer process parameters on the electrical and mechanical properties of screen-printed silver layers. Such layers are a fundamental element of many different conductive applications as they provide a way to interconnect elements or transfer power or data. Among their many purposes, they can serve as conductors for other electronics, heaters [19], RFID antennas [21], or other functional layers. The resistance of screen-printed materials can change during the heat transfer process [20], and due to the need for specific resistances for many applications, it is essential to know the exact changes after the heat transfer process. Using parameters suggested by suppliers may not always yield optimal mechanical or electrical properties due to interactions between the layers of multilayered printed electronics.

This work investigates how changes in heat transfer process parameters influence screen-printed silver layers’ electrical and mechanical properties. The results from this study will help to understand the heat transfer process better and be a basis for further research into this topic.

## 2. Materials and Methods

### 2.1. Materials

Three types of silver composites were selected for this study. One of the composites, Loctite EDAG 725A, was commercially acquired from Henkel in Germany. The other two were prepared using AX 20LC silver flakes from Amepox in Poland with an average size of particles of 2–4 µm and a paraffin coating, with two different vehicles. The first composite was based on a copolymer acquired from Novelinks in Poland, while the second composite was based on thermoplastic polyurethane (TPU) Elastollan 1170A purchased from BASF in Germany. For the TPU-based composite, commercially available TPU with a density of 1.18 g/cm^3^ was dissolved in a mixture of Tetrahydrofuran (THF) and Dimethylformamide (DMF), combined in a 1:2 ratio. The silver composites were then screen-printed on a matte 2C-CP transfer film acquired from the Texo Trade Services in the Netherlands and printed over with SPTN Sicoplast plastisol paint from SICO in Poland. The entire pattern was then heat transferred onto 120 g/m^2^ 100% polyester cloth.

### 2.2. Preparations

To produce the TPU-based vehicle, the polymer was dissolved in a 1:2 THF and DMF mixture with a magnetic mixer for 4 h at a temperature of 40 °C. This resulted in a vehicle containing 20 wt.% of TPU. Two composites, TPU-Ag and Novel-Ag, were produced by mixing silver flakes with a TPU-based vehicle and a vehicle from Novelinks, respectively, to acquire 70 wt.% printing pastes. The measured viscosity values of composites measured at a shear rate of 50 1/s were 2.64 Pa·s for TPU-Ag and 6.25 Pa·s for Novel-Ag.

**Figure 1:**
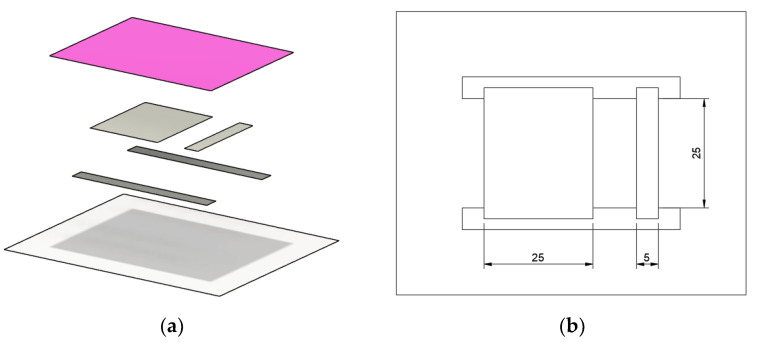
Design of the printed test pattern: (**a**) From the bottom: transfer print, electrical contacts screen printed with EDAG 725a, test surfaces printed with analyzed materials, isolating layer printed with plastisol paint; (**b**) top view without isolating layer showing dimensions of test surfaces.

Everything was screen printed using 68T polymer mesh screens on an Aurel C920 screen printer. Squeegee pressure was set to around 64 N and printing speed to 150 mm/s. After the initial tests, electric contacts were screen printed with Loctite EDAG 725A, as this composite was found to be transferable in a broader temperature range. Subsequently, all composites (TPU-Ag, Novel-Ag, and EDAG 725A) were screen-printed in two patterns: 5 by 25 mm for electrical conductivity testing and 25 by 25 mm for mechanical tests. To ensure good heat transfer and electric isolation, an additional layer of plastisol paint was printed on top of the composite layers (Figure 1). Each layer was dried for 20 min at 120 °C to ensure proper curing.

The heat transfer process was carried out using a Secabo TC7 heat press with variable time (30, 60, 90, 120 s), temperature (ranging from 150 to 220 °C), and a force of 150 g/cm^2^. The chosen range of time and temperatures was based on the minimal requirements of the plastisol paint as stated by the producer (30 s and 150 °C), as well as the maximum values determined by the maximum operating temperature of the heat press and the suggested maximum operating time.

### 2.3. Methods

Apparent viscosity was measured with the use of an R/S Plus Rheometer (Brookfield Engineering, Middleboro, MA, USA) equipped with an RCT-50-2 spindle using the cone-plate method. The measurement procedure was an increase of rotation speed from 0 to 500 rpm in 200 s. All measurements were performed at a controlled temperature of 25 ± 1 °C. The data were analyzed with Brookfield Rheo 3000 software (Brookfield Engineering, Middleboro, MA, USA). Viscosity was expressed in Pa∙s. The viscosities of the tested inks at the shear rate of ~50 s^−1^ were compared.

Resistances were measured using a two-point method using a 6½ digit Keysight 32251A laboratory multimeter.

The bend test was conducted using a Cometech QC-Tech M2 tensile machine. Sets of ten samples were bent with the conductive layer on the inside to a radius of 5 mm. The procedure was repeated for 1000 cycles. Electrical measurements were performed before and after the test.

The hand abrasion test was performed using an in-house tester simulating a finger moving over the surface of the transferred samples. Each sample was tested through increments of 100 cycles and visually inspected after each increment.

SEM imaging was done with HITACHI SU8230 at 10 kV using secondary electron detection.

The surface thickness was measured with a BRUKER DektakXT profilometer.

## 3. Results and Discussion

To ensure the viability of screen-printing with the prepared pastes, their rheological properties were measured using the cone-plate method. The measured viscosity values were 2.64 Pa·s and 6.25 Pa·s for TPU-Ag and Novel-Ag, respectively. Values were collected for a shear rate of 50 1/s. These viscosity values indicated that there would be no issues with screen-printing, as the pastes exhibited suitable flow characteristics.

**Figure 2:**
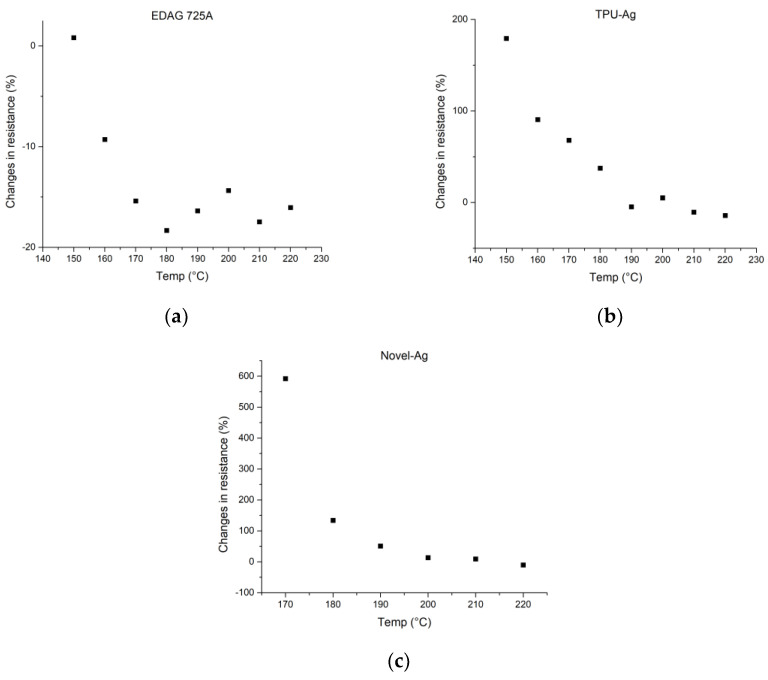
Preliminary measurements used to determine the viable range. Figures display the relationship between temperatures of the heat transfer process at a time of 60 s and % changes in resistance before and after heat transfer for composites: (**a**) EDAG 725A; (**b**) TPU-Ag; (**c**) Novel-Ag.

After screen-printing, each composite was subjected to heat transfer at a temperature range of 150–220 °C for 60 s to determine a viable production range. The criterion for determining viability was an increase in resistance no higher than 100% of the initial value before screen-printing. All samples were measured before and after the process. The resulting changes in resistance from the first series of samples are presented in Figure 2 and the exact resistance values that were used to determine those changes are presented in Appendix A in Table A1.

**Figure 3:**
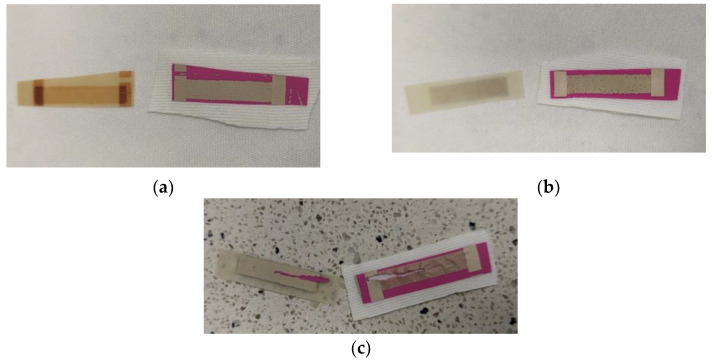
Photos representing issues with heat transfer for each of silver composites: (**a**) EDAG 725A—burnt layer left on a release foil at 190 °C; (**b**) TPU-Ag—poor heat transfer quality at 150 °C; (**c**) Novel-Ag—lack of transfer at 160 °C.

Based on the results obtained from the experimental tests, specific temperature ranges were chosen for each composite tested. The Loctite EDAG 725A composite showed successful transferability in the temperature range of 150–220 °C. However, it was observed that at temperatures above 190 °C, the silver composite left a burnt layer on the release foil, as shown in Figure 3a. As a result, the final chosen range for EDAG 725A was narrowed down to 150–180 °C to avoid the issue of burnt layers.

For the TPU-Ag composite, it was found that the composite did not release correctly at 150 °C, as evidenced by Figure 3b, and exhibited a significant increase in resistance of 190% at that temperature. Therefore, the temperature range for the TPU-Ag composite was adjusted to 160–190 °C to ensure the proper release and transfer of the composite.

The Novel-Ag composite did not transfer at all at 150 °C and 160 °C, as shown in Figure 3c. Moreover, it exhibited a high increase in resistance at 170 °C. As a result, a temperature range of 180–210 °C was chosen for the transfer of Novel-Ag to achieve optimal results.

**Figure 4:**
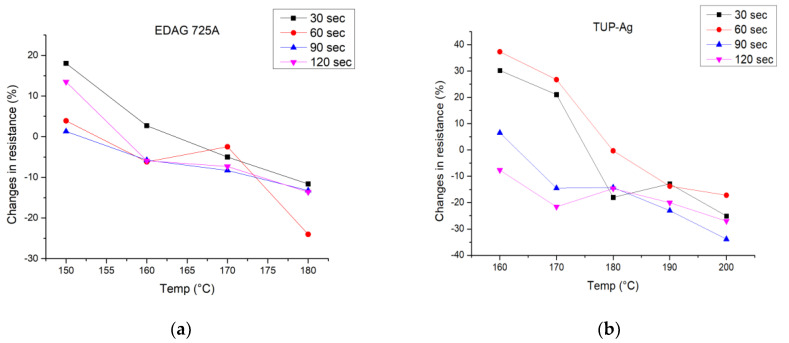

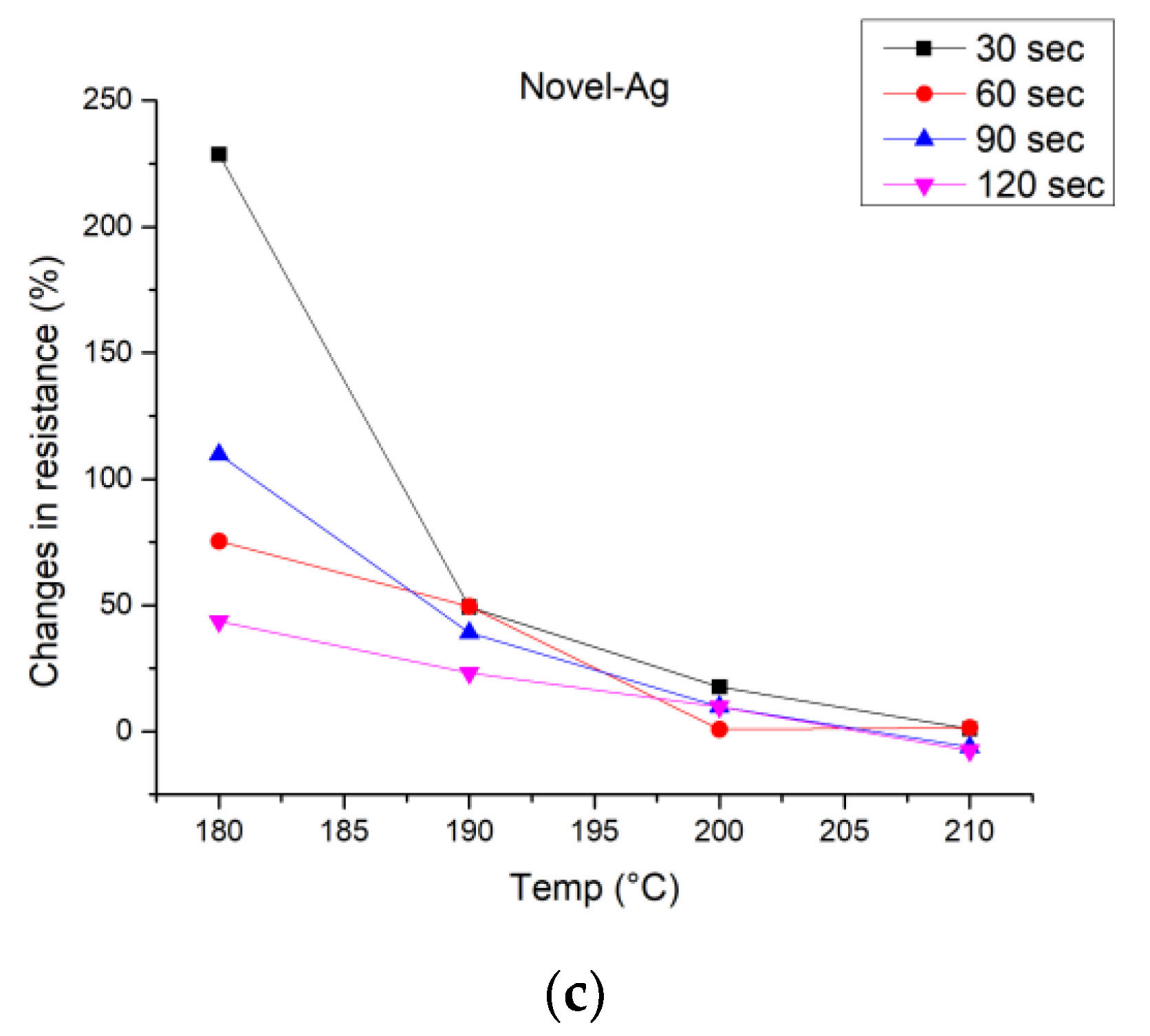
Results representing a relationship between the temperature of the heat transfer process and changes in resistance after this process for 30 s, 60 s, 90 s, and 120 s using silver composites: (**a**) EDAG; (**b**) TPU-Ag; (**c**) Novel-Ag.

Once viable production windows were established for each composite based on the previous heat transfer tests, further measurements were conducted to assess the effect of different heat transfer times. Heat transfer times of 30 s, 60 s, 90 s, and 120 s were chosen for evaluation. Three samples were measured before heat transfer and after the heat transfer process. Changes in the resistances between those values are presented in Figure 4 and the average resistances of those measurements are presented in Appendix A in Table A2.

The results presented in Figure 3 clearly demonstrate that each composite exhibits lower resistance changes with an increase in temperature, with EDAG 725A and TPU-Ag showing a reduction in initial resistance by 24% and 23%, respectively, at higher temperatures. Prints made with EDAG 725A remain stable for all transfer times but transferring for 60 s allows for low resistances even at lower temperatures. For TPU-Ag, there is a significant difference between the results acquired with times of 30 s, 60 s, 90 s, and 120 s, with lower resistances observed for longer times at 160 °C and 170 °C and diminishing at 180 °C and 190 °C. For Novel-Ag, apart from the results at 180 °C, differences in resistance changes are not as significant, slightly favoring longer heat transfer times.

With the resistance changes known for each composite, determining the influence of changes in time and temperature on mechanical properties was needed. For that, three points for each sample were chosen. The first point with the lowest temperature and a low time, such that the increase in resistance is small. The second point was chosen in the middle of the range and the third point was chosen with the highest temperature, a high decrease in resistance, and a lower time if possible. For EDAG 725A, they were: 150 °C and 60 s, 170 °C and 30 s, 180 °C and 60 s; for TPU-Ag: 160 °C and 30 s, 180 °C and 30 s, 200 °C and 30 s; for Novel-Ag: 180 °C and 60 s, 200 °C and 60 s, 210 °C and 90 s.

**Figure 5:**
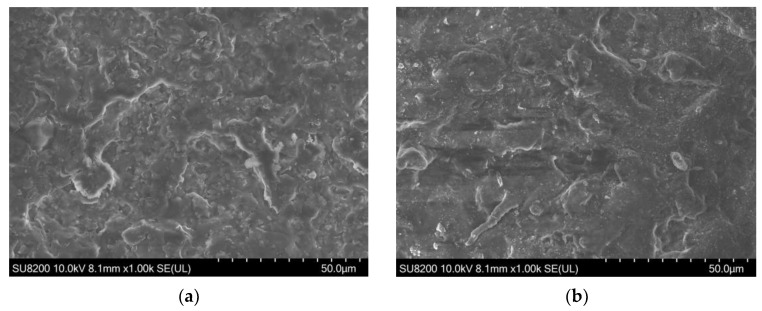

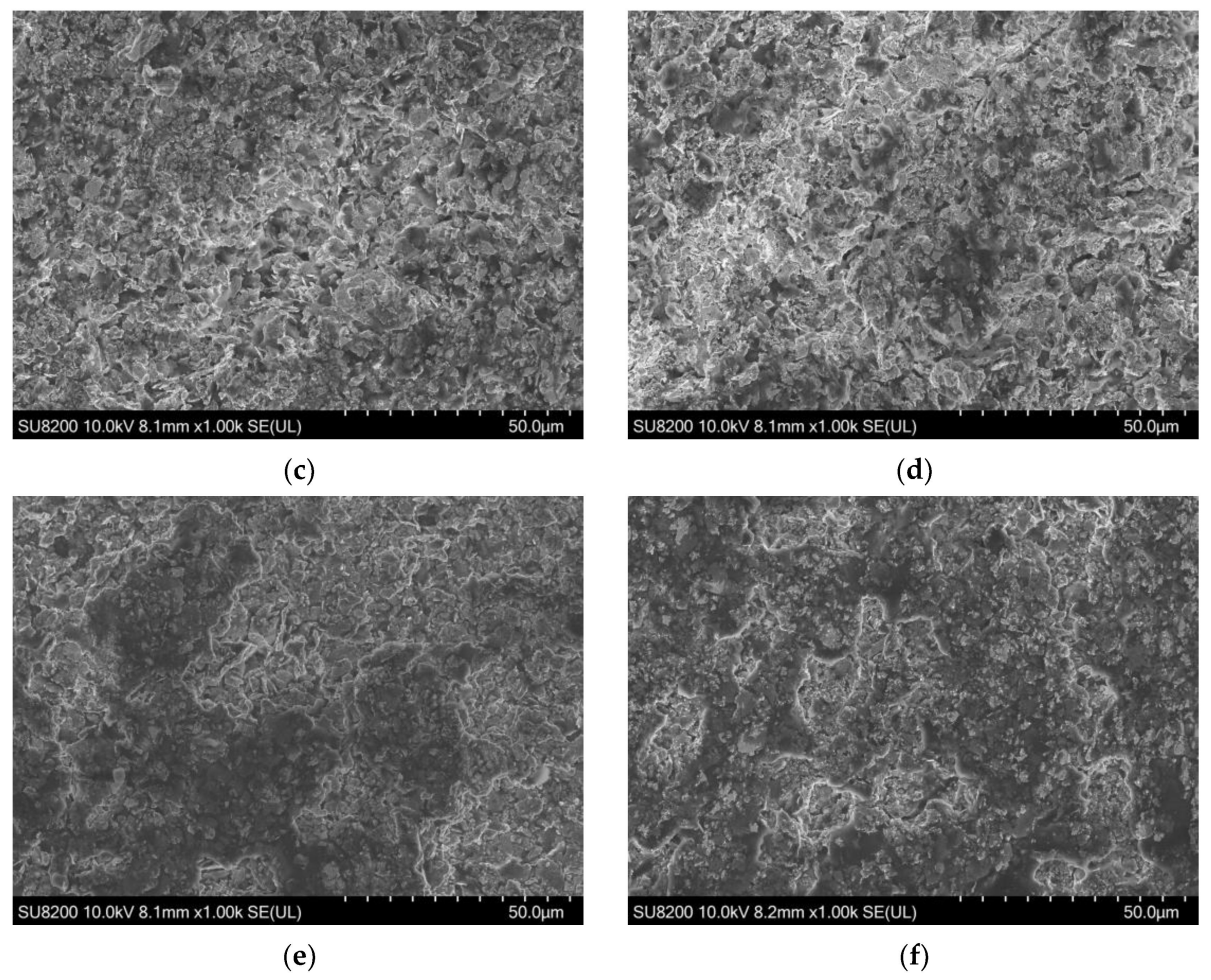
SEM images of heat-transferred layers at different times and temperatures showcasing the increased amount of polymers in the top layers of a composite: (**a**) EDAG 725A heat transferred at 150 °C and 60 s; (**b**) EDAG 725A heat transferred at 180 °C and 60 s; (**c**) TPU-Ag heat transferred at 160 °C and 30 s; (**d**) TPU-Ag heat transferred at 200 °C and 30 s; (**e**) Novel-Ag heat transferred at 180 °C and 60 s; (**f**) Novel-Ag heat transferred at 210 °C and 90 s.

To observe differences in the microstructure of heat-transferred layers, SEM images were captured. Such a procedure was performed for every composite at their chosen measuring points. Sample SEM images of the first and third points displaying increased amount of polymer on a surface of heat-transferred layers are presented in Figure 5.

With the increased pressure and temperature of heat transfer, the microstructure of the printed layer changed. The polymer matrix that holds together silver particles softened and with pressure from the heat transfer press, it allowed for a flow of the polymer to the upper layers of the composite and decreased the distances between single silver flakes in deeper parts of the composite, lowering overall resistance. Those changes were noticeable in the SEM images provided. For EDAG 725A, silver flakes stopped being noticeable beneath the polymer layer, and for TPU-Ag and Novel-Ag, the sizes and density of the polymer visible as shadowed portions increased.

To further test this hypothesis, the thickness of the layers was measured. For this, samples on foil were only heat pressed and not transferred, so they could be measured with a profilometer. Those findings are presented in Table 1. 

For all composites, there was a noticeable increase in the height of the layer. With an increased amount of polymer at the top of the composite, it could polymerize in bigger agglomerates, allowing for a structure with a higher volume and increasing overall thickness. 

Further tests were carried out on the selected samples to determine their mechanical strength. Two parallel tests were carried out, namely the bend test and the hand abrasion test. In the bend test, six samples from each set were bent to a 5 mm radius with conducting layers on the inside of the bend for 1000 cycles, and the changes in resistance before and after the test of the transferred prints are displayed in Table 2.

When it comes to TPU-Ag and Novel-Ag composites, there is no discernible change in resistance, indicating that the heat transfer parameters do not significantly influence their mechanical properties. However, for EDAG 725A, there is a notable increase in resistance change with an increase in temperature. This suggests that the polymer used in this composite becomes more brittle at higher temperatures, resulting in irreversible changes during the fatigue test. The increase in resistance may be indicative of structural changes or damage to the silver layer. SEM images were taken after the bend test. There were no visible differences in the surface of the composite between samples before and after the test, suggesting that the resulting changes appeared in the lower part of the composite.

**Figure 6:**
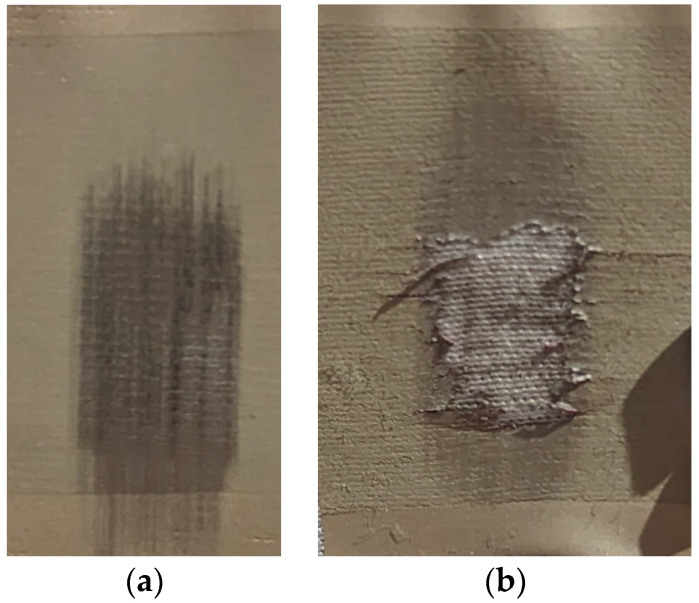
Exemplar results of the hand abrasion test (**a**) Visible breaks in a surface of the EDAG 725A sample produced with parameters of 170 °C and 30 s after 750 cycles; (**b**) Delamination of a surface layer of the TPU-Ag sample produced with parameters of 160 °C and 30 s after 300 cycles.

The second mechanical test conducted was the hand abrasion test, which involved rubbing a cloth-covered synthetic finger against the surface of the heat-transferred print in a repeating pattern. The test was carried out in increments of 50 cycles until visible fractures in the structure of the silver composite appeared, indicating wear and damage (Figure 6a). Notably, for samples with the lowest heat transfer parameters, complete delamination of an entire layer was observed after an additional 100 cycles of the hand abrasion test after the appearance of the first visible fractures (Figure 6b).

For each of the samples, three tests were conducted, and the average values of the results are displayed in Table 3.

The results reveal a clear mechanical advantage in using higher temperatures and longer times during heat transfer. Increasing both of these parameters led to significant improvements in adhesion strength between the printed composite and the substrate. Notably, at low heat transfer parameters, the mechanical endurance of the printed composites is not sufficient for practical use in wearable electronics, as evidenced by the observed wear and delamination in the hand abrasion test. These findings underscore the importance of carefully selecting and optimizing the heat transfer parameters to achieve the desired mechanical properties and durability of printed composites for specific applications, such as wearable electronics, where mechanical performance is crucial for long-term functionality and reliability.

## 4. Conclusions

The experiments conducted in this study revealed the significant impact of heat transfer process parameters on the electrical and mechanical properties of printed composites for wearable electronics. The results highlighted that increasing the time and temperature during heat transfer can lead to improved adhesive strength and lowered resistance, although it was observed that not all materials maintained their mechanical parameters after the process, as demonstrated by the bend test. Furthermore, the study underscored the importance of tailoring the heat transfer parameters for each silver composite, emphasizing the need for careful optimization to achieve desired performance.

The findings of this research provide valuable insights into the feasibility of using heat transfer with screen printing to produce electronics on textiles. It serves as a promising starting point for further research in this area, opening up opportunities for exploring additional factors that could further enhance the mechanical properties, electrical performance, and durability of printed composites for wearable electronics. The ability to fine-tune the mechanical and electrical properties of materials by changing heat transfer parameters can enable the fabrication of functional printed composites with tailored properties for specific applications in wearable electronics, such as smart clothing, wearable sensors, and biomedical devices.

In summary, the results of this study contribute to the understanding of the relationship between heat transfer parameters and the electrical and mechanical properties of printed composites for wearable electronics. The findings provide guidance for researchers and practitioners in the field of printed electronics and pave the way for future research and development in advancing the fabrication of functional and reliable wearable electronics by using heat transfer processes.

## Figures and Tables

**Table 1:** Changes in the thickness of the heat-pressed layer at different times and temperatures.

Composite	Temperature [°C]	Time [s]	Thickness [µm]
EDAG 725A	150	60	6.07
	170	30	7.09
	180	60	7.42
TPU-Ag	160	30	3.84
	180	30	6.83
	200	30	8.88
Novel-Ag	180	60	11.3
	200	60	11.08
	210	90	11.88

**Table 2:** Changes in resistance after performing a bend test for 1000 cycles to a radius of 5 mm.

Composite	Temperature [°C]	Time [s]	Average Resistance before Bend Test [mΩ]	Average Resistance after Bend Test [mΩ]	Change in Resistance [%]
EDAG 725A	150	60	167.17 ± 14.60	388.00 ± 66.70	130.97 ± 26.98
	170	30	153.17 ± 7.95	574.83 ± 77.60	276.89 ± 57.34
	180	60	121.33 ± 5.99	506.83 ± 91.77	318.11 ± 77.98
TPU-Ag	160	30	148.50 ± 16.89	153.50 ± 15.50	3.59 ± 3.92
	180	30	104.00 ± 5.94	105.33 ± 5.47	1.34 ± 2,45
	200	30	94.67 ± 4.46	96.17 ± 6.64	1.59 ± 5.13
Novel-Ag	180	60	84.34 ± 7.50	89.00 ± 10.78	5.96 ± 13.01
	200	60	70.84 ± 3.44	71.00 ± 5.23	0.14 ± 3.34
	210	90	70.62 ± 6.20	70.83 ± 5.70	0.36 ± 3.23

**Table 3:** Table representing the average number of cycles of the hand abrasion test needed to create visible fractures in the surface of transferred prints.

Composite	Temperature [°C]	Time [s]	Average Number of Cycles
EDAG 725A	150	60	166.67 ± 23.57
	170	30	716.67 ± 84.98
	180	60	2466.67 ± 124.72
TPU-Ag	160	30	216.67 ± 6236
	180	30	1250.00 ± 70.71
	200	30	2066.67 ± 94.28
Novel-Ag	180	60	250.00 ± 40.82
	200	60	566.67 ± 62.36
	210	90	2100.00 ± 81.65

## Data Availability

The data that support the findings of this study are available from the corresponding author upon reasonable request.

## Appendix A

This appendix contains tables displaying values used for discerning % changes of the resistances.

**Table A1:** Resistance values before and after heat transfer process collected at different temperatures at 60 s heat transfer time.

Composite	Temperature [°C]	Resistance before Heat Transfer [mΩ]	Resistance after Heat Transfer [mΩ]	Change in Resistance [%]
EDAG 725A	150	369	372	0.81
	160	366	332	−9.29
	170	396	335	−15.40
	180	393	321	−18.32
	190	409	342	−16.38
	200	390	334	−14.36
	210	412	340	−17.48
	220	405	340	−16.05
TPU-Ag	150	139	388	179.14
	160	190	362	90.53
	170	125	210	68.00
	180	152	209	37.50
	190	192	183	−4.69
	200	197	207	5.08
	210	162	145	−10.49
	220	177	152	−14.12
Novel-Ag	170	159	1100	591.82
	180	155	363	134.19
	190	148	271	83.11
	200	157	178	13.38
	210	151	165	9.27
	220	157	141	−10.19

**Table A2:** Resistance values before and after heat transfer process collected at different temperatures and different times of heat transfer.

Composite	Temperature [°C]	Time [s]	Average Resistance before Heat Transfer [mΩ]	Average Resistance after Heat Transfer [mΩ]	Change in Resistance [%]
EDAG 725A	150	30	401.67 ± 15.28	475.333 ± 83.39	18.02
	150	60	394.00 ± 23.26	409.67 ± 32.65	3.90
	150	90	425.00 ± 23.43	429.67 ± 8.50	1.29
	150	120	404.33 ± 10.69	458.67 ± 8.50	13.47
	160	30	397.67 ± 14.74	409.00 ± 56.40	2.70
	160	60	395.33 ± 25.58	371.33 ± 34.59	−6.19
	160	90	389.33 ± 35.35	365.67 ± 13.01	−5.76
	160	120	381.00 ± 13.45	358.00 ± 7.81	−5.91
	170	30	358.67 ± 77.57	313.50 ± 54.22	−5.01
	170	60	314.67 ± 70.55	301.67 ± 29.96	−2.47
	170	90	353.67 ± 6.66	324.33 ± 5.51	−8.29
	170	120	368.33 ± 23.07	341.00 ± 14.18	−7.34
	180	30	398.67 ± 19.86	352.00 ± 9.17	−11.63
	180	60	410.00 ± 16.09	311.67 ± 59.55	−24.00
	180	90	416.67 ± 7.57	361.67 ± 25.42	−13.22
	180	120	421.33 ± 27.59	363.67 ± 18.01	−13.62
TPU-Ag	160	30	193.00 ± 15.12	251.3319.75	30.22
	160	60	201.33 ± 8.99	273.33 ± 62.74	37.33
	160	90	219.33 ± 23.13	230.33 ± 20.95	6.55
	160	120	207.33 ± 16.11	190.67 ± 6.34	−7.65
	170	30	183.33 ± 16.76	222.33 ± 25.38	21.06
	170	60	172.33 ± 40.53	204.67 ± 10.50	26.73
	170	90	208.33 ± 9.18	178.33 ± 15.33	−14.48
	170	120	198.00 ± 9.90	155.33 ± 25.82	−21.59
	180	30	229.67 ± 5.56	188.00 ± 14.45	−18.00
	180	60	198.33 ± 35.42	188.33 ± 14.61	−0.29
	180	90	227.33 ± 16.50	193.33 ± 16.82	−14.25
	180	120	206.67 ± 5.79	176.33 ± 3.40	−14.64
	190	30	201.33 ± 8.34	175.33 ± 10.78	−12.84
	190	60	217.00 ± 17.80	186.00 ± 6.48	−13.73
	190	90	233.67 ± 6.60	179.67 ± 8.99	−23.02
	190	120	214.00 ± 9.42	171.00 ± 4.55	−20.00
	200	30	228.67 ± 1.70	171.33 ± 3.30	−25.06
	200	60	220.00 ± 16.87	179.67 ± 19.75	−17.18
	200	90	235.67 ± 8.96	155.67 ± 4.92	−33.86
	200	120	228.00 ± 3.74	166.33 ± 1.25	−27.019
Novel-Ag	180	30	146.33 ± 1.25	1535.00 ± 1495.04	228.68
	180	60	149.67 ± 3.86	264.00 ± 70.27	75.37
	180	90	148.67 ± 8.18	308.67 ± 44.68	109.72
	180	120	163.33 ± 6.24	234.33 ± 12.66	43.67
	190	30	151.25 ± 12.03	240.75 ± 41.48	49.31
	190	60	150.00 ± 2.16	224.00 ± 33.24	49.57
	190	90	151.33 ± 10.50	211.33 ± 28.55	39.12
	190	120	163.00 ± 13.14	198.67 ± 19.69	23.19
	200	30	149.67 ± 7.85	175.67 ± 9.98	17.62
	200	60	156.67 ± 0.47	158.00 ± 14.24	0.84
	200	90	144.33 ± 3.30	158.33 ± 7.85	9.88
	200	120	152.33 ± 1.70	167.33 ± 18.12	9.79
	210	30	158.33 ± 2.87	160.00 ± 7.12	1.01
	210	60	144.33 ± 4.19	146.67 ± 12.97	1.45
	210	90	152.33 ± 7.13	143.33 ± 14.08	−6.13
	210	120	157.67 ± 5.79	146.67 ± 3.68	−7.55
